# Institutional Design of Forest Landscape Restoration in Central Togo: Informing Policy-making through Q Methodology Analysis

**DOI:** 10.1007/s00267-025-02137-x

**Published:** 2025-03-03

**Authors:** Hamza Moluh Njoya, Kossi Hounkpati, Kossi Adjonou, Kouami Kokou, Stefan Sieber, Katharina Löhr

**Affiliations:** 1 Leibniz Centre for Agricultural Landscape Research (ZALF), Sustainable Land Use in Developing Countries, 15374 Müncheberg, Germany; 2 Department of Agricultural Economics, Faculty of Life Sciences, Humboldt Universität zu Berlin, 10099 Berlin, Germany; 3 Department of Rural Socio-Economics and Agricultural Extension, Faculty of Agronomy and Agricultural Sciences of the University of Dschang, 222 Dschang, Cameroon; 4 Forest Research Laboratory, Climate Change Research Centre, University of Lomé, 1515 Lome, Togo; 5 Urban Plant Ecophysiology, Humboldt Universität zu Berlin, 10099 Berlin, Germany

**Keywords:** Forest landscape restoration, Stakeholder perceptions, Policy design, Q methodology, Community engagement

## Abstract

Forest landscape restoration (FLR) is a promising tool for restoring ecological functionality and improving human well-being in degraded landscapes. The success of FLR efforts depends on the interests, perceptions, and actions of local communities, extension services, Non-Governmental Organizations, and policymakers. While much research focuses on the direct ecological and economic impacts of FLR, limited attention has been given to how stakeholder perceptions influence the design and implementation of restoration efforts. Understanding these perspectives is crucial for shaping effective policy interventions and ensuring long-term FLR success. This study uses Q methodology to examine stakeholder viewpoints on key considerations and priorities for designing and implementing FLR in Tchamba Prefecture, Togo. The analysis reveals three distinct perspectives: (1) Incentive-Driven Restoration, emphasizing financial incentives and private-sector partnerships as essential for FLR success; (2) Comprehensive and Collaborative Restoration, advocating for an inclusive, multidisciplinary approach that integrates community participation and long-term monitoring; and (3) Incentive-Driven Community Restoration, highlighting the importance of economic incentives in fostering local engagement. Across all perspectives, stakeholders strongly agreed on the importance of biodiversity conservation, stakeholder involvement, and conflict resolution in land use. The findings underscore the need to tailor FLR policies to local contexts and stakeholder preferences, suggesting that flexible, participatory approaches can enhance sustainability and effectiveness. This study contributes to developing inclusive, adaptive restoration policies and highlights the need to integrate behavioural insights into policy-making to foster long-term stakeholder engagement.

## Introduction

Over the past decade, various actors have proposed new solutions to address environmental and social problems associated with global land degradation (Mansourian et al., 2017a). One such approach, forest landscape restoration (FLR) seeks to restore degraded landscapes to enhance biodiversity, ecosystem services, and livelihoods (Eshetu et al., 2024; Mansourian et al., 2024). By tackling the root causes of deforestation, FLR promotes sustainable land management. Recognizing this urgency, many Sub-Saharan African (SSA) countries have committed to restoring over 100 million hectares of degraded land by 2030 under the African Forest Landscape Restoration Initiative (AFR100), aligning with the Bonn Challenge’s global target of 350 million hectares (Djenontin et al., 2022; Owusu et al., 2021). However, population growth, urbanization, and unsustainable consumption continue to place immense pressure on natural resources (Lukina, 2020). Globally, 30% of potential forests have been lost, and another 20% degraded, with two billion hectares affected by soil degradation (Gibbs and Salmon, 2015). The AFR100 and Bonn Challenge commitments highlight FLR’s critical role in achieving sustainable development goals, including poverty reduction, food security, and climate change mitigation (Pirelli et al., 2020). Additionally, FLR supports livelihoods, social cohesion, and conflict resolution in communities worldwide (Ullah, 2024).

Togo, covering 56600 km², is an excellent example of strategic restoration planning within the AFR100. This initiative responds to severe forest and land degradation driven by insufficient forest management and unsuitable farming practices, leading to soil fertility decline (Kombate et al., 2022). Togo experiences an annual land degradation rate of 4.14%, equivalent to 23,490 hectares. The country has committed to restoring 80% of degraded land by 2030 and limiting intact land degradation to 2%, aiming to restore 1.4 million hectares of forest landscape, which constitutes 10.20% of the nation’s forest cover (MERF, 2018; Pirelli et al., 2020; Stanturf and Mansourian, 2020). Togo’s FLR efforts prioritize local smallholder farmers and resource users, focusing on sustainable land management practices through multifaceted approaches outlined in the National Reforestation Programme (PNR). The goal is to enhance ecosystem services, including biomass-derived energy sources, wood resources, non-timber forest products (NTFPs), water management, carbon sequestration, and preservation of cultural and religious values (McBreen and Jewell, 2023). These efforts are critical for addressing declining ecosystem services, livelihoods, food security, and biodiversity in the environmentally degraded Tchamba Prefecture(Hounkpati et al., 2024a). While these national efforts span across the country, some regions, such as Tchamba Prefecture, face particularly severe degradation challenges, necessitating targeted restoration interventions.

Although other regional prefectures face similar environmental challenges, Tchamba Prefecture stands out due to the severity of land degradation, making it a priority site for FLR intervention. To support Togo’s commitment to the Bonn Challenge, Tchamba was selected for targeted restoration efforts. Five of its ten cantons (Affem-Boussou, Alibi 1, Bago, Goubi, and Koussountou) were chosen for the Global Project on FLR, implemented by the Deutsche Gesellschaft für Internationale Zusammenarbeit (GIZ Forests4Future, F4F). This initiative focuses on community forestry, participatory mapping, and assessing the potential for wood and non-wood forest products. Under contract with GIZ-F4F-Togo, the Deutsche Forst Service GmbH (DFS) began field activities in 2020. These five cantons represent the F4F intervention zone, where the project aims to restore productive forests and strengthen governance (Hounkpati et al., 2024b). The selection builds on previous efforts by ProREDD and the International Forest Policy (IWP), which helped manage four multipurpose community forests spanning 15,838 hectares (Hounkpati et al., 2024b). Togo aims to reforest one billion trees and expand forest cover from 24.24% to 25% by 2030 and 30% by 2050 (MERF, 2016), Against this backdrop, this study examines stakeholder perspectives on FLR design and implementation, essential for advancing Togo’s national restoration agenda.

In 2000, a consortium of 30 scientists first defined FLR as “a planned process aimed at restoring ecological integrity and enhancing human well-being in deforested or degraded landscapes.” (Mansourian et al., 2017b). More recently, McBreen and Jewell (2023) redefined FLR as the long-term effort to restore ecosystem functionality and improve human well-being across deforested and degraded landscapes. FLR aims to rehabilitate forests while balancing human needs with ecological goals.These definitions raise the question of the objective of FLR. As Mansourian (2017) emphasizes, FLR does not seek to turn entire landscapes into forests but rather to improve forest quality to benefit both people and biodiversity. It is a holistic and dynamic approach that enhances biodiversity, ecosystem services, and climate resilience (Chazdon and Guariguata, 2016; Yusof et al., 2023). FLR integrates ecological, social, and economic objectives, fostering synergies between biodiversity conservation, climate change mitigation, and sustainable development (Stanturf, 2021). Successful FLR requires active participation from local communities, governments, Non-Governmental Organizations (NGOs), and the private sector to address the root causes of degradation and promote sustainable land management (Ahammad et al., 2023). Additionally, FLR incorporates traditional knowledge and participatory approaches that empower communities and promote social equity (Maniraho et al., 2023).

In a landscape context, viewpoints influence people’s actions and behaviour toward natural resource management (Bayala, 2023). Stakeholders are crucial in achieving FLR goals, making it essential to understand their concerns, motivations, and expectations (Cebrián-Piqueras et al., 2023). Improving FLR governance requires addressing stakeholders’ varied interests and challenges in restoration efforts. Governance challenges such as land tenure security, policy inconsistencies, and institutional coordination significantly impact FLR success (Mansourian, 2017). According to Mansourian (2017), the bidirectional relationship between large-scale forest restoration and governance underscores the need for a comprehensive and adaptive approach to FLR. The governance approach itself influences stakeholder engagement—conservation initiatives perceived as inclusive and beneficial encourage participation, while those seen as restrictive or unfair can lead to resistance and disengagement (Bayala, 2023). Additionally, access to benefits or fear of reliving past failures shapes local participation decisions (Gilli et al., 2020). Effective collaborative landscape governance can only be achieved if local actors perceive governance structures positively. Conversely, negative perceptions may lead communities to discontinue collaboration, undermining co-management and sustainable resource use efforts (Carmenta et al., 2017). Thus, integrating stakeholder perceptions into conservation strategies is crucial, as these views significantly impact social and ecological outcomes (Bennett, 2016; Schulze and Matzdorf, 2023). This study examines local stakeholder perceptions of FLR governance in Tchamba Prefecture, Central Togo, identifying consensus entry points to inform more inclusive and adaptive FLR implementation strategies.

Globally, research has studied the FLR concept and landscape approaches. Such evidence includes the contribution of FLR in combating soil erosion in the Lake Abaya catchment, Southern Ethiopia (Eshetu et al., 2024); the FLR and its impact on social cohesion, ecosystems, and rural livelihoods in Pakistan (Ullah, 2024); The potential for FLR in the Amazon (da Silva et al., 2023); The cost-benefit analysis of China’s FLR program (Wang et al., 2023); Social learning and promotion of forest restoration in a semi-arid landscape in North Africa (Derak et al., 2024). However, while these studies predominantly focus on the ecological and economic aspects of FLR, other works, such as those by Bayala (2023); Mansourian (2017); Schulze and Matzdorf (2023), have delved into governance aspects related to natural resource management. Regardless of these efforts, there is still a gap in understanding the nuanced viewpoints of local stakeholders on how FLR governance systems should be effectively implemented, indicating the need for further investigation to inform more inclusive and effective policy frameworks for the long-term success of restoration efforts.

Therefore, the present study aims to identify consensus entry points for the institutional design of FLR initiatives in Togo. The main question guiding this study is, “how do stakeholder perceptions inform the effective implementation of FLR governance systems in Togo”? Specifically, (i) Who are the main stakeholders involved in FLR in the Tchamba Prefecture of central Togo? (ii) What are the key perspectives and areas of consensus and divergence among stakeholders that can guide the development of effective and inclusive restoration policies? To better capture stakeholders’ perceptions on FLR design in Togolese forest and agricultural landscapes, we interviewed experts about the ecological and conservation focus, community and local engagement, governance and policy-making, funding and incentives. Our data come from a qualitative expert interview (N = 15 experts) conducted in August- September 2023 in Tchamba Prefecture, Togo. We use the Q methodology to capture the stakeholders’ viewpoints in FLR. The Q methodology is a distinctive semiquantitative technique for exploring human perspectives (Zabala et al., 2018). Bayala (2023); Schulze and Matzdorf (2023) used such a semiquantitative method to study the stakeholder’s engagement in conservation and environmental management.

Our research addresses gaps in current knowledge regarding forest and agricultural landscape restoration, providing valuable insights into stakeholder perceptions of governance effectiveness in FLR. Policymakers could use such an approach to develop more targeted and inclusive programs, enhancing the efficacy of conservation measures. By recognizing the importance of multiple stakeholders and considering behavioural factors in policy-making processes, our study underscores the need for collaborative and holistic approaches to FLR governance.

## Methodology

### The Q Methodology

**Fig. 1:**
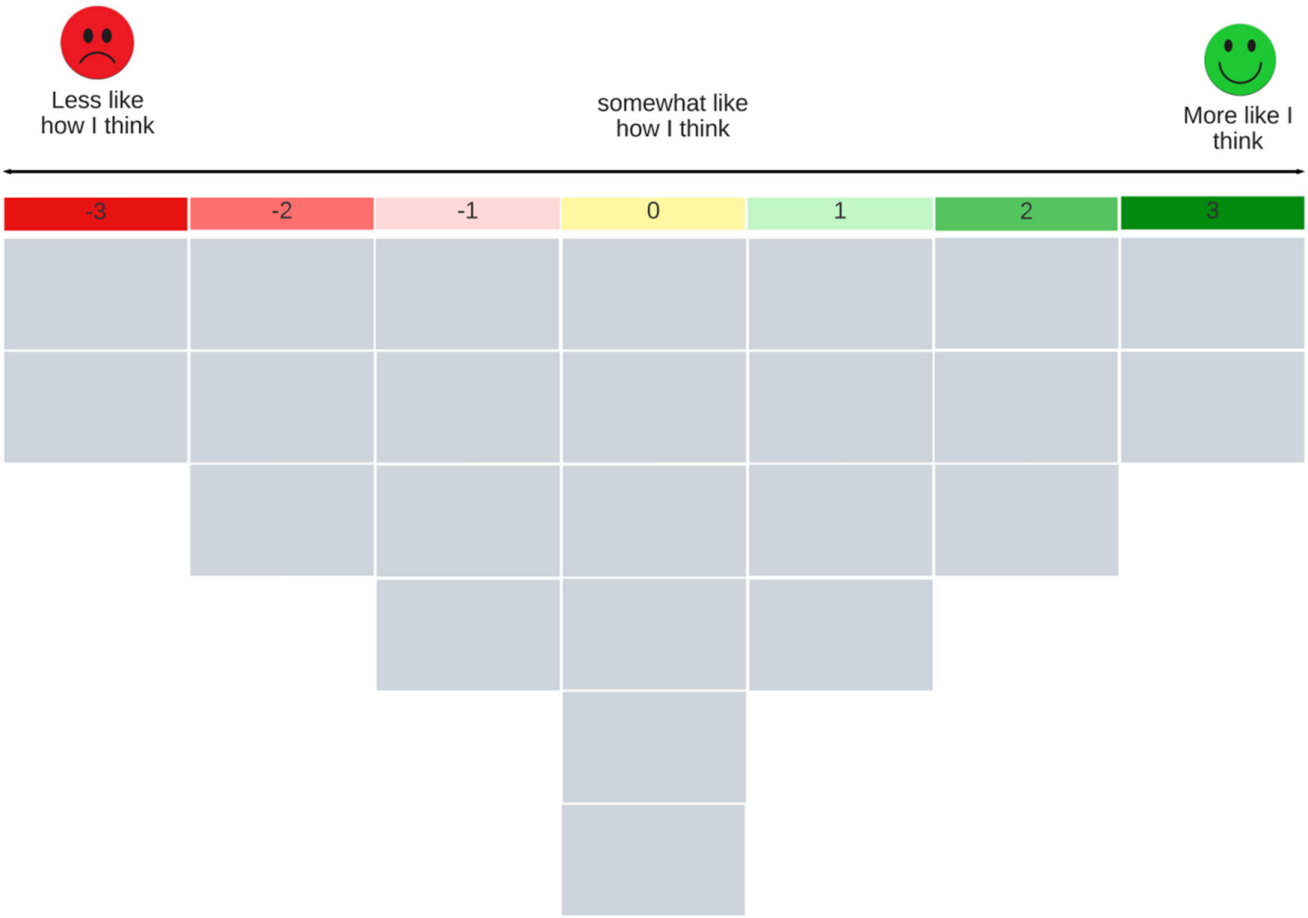
The Q-sort grid applied in this research shows the level of agreement or disagreement with the statements, following a quasi-normal distribution. The numbers along the top are values used for analysis and are not seen by participants. Source: adapted from Schulze and Matzdorf (2023)

This study uses Q methodology to identify common concerns and entry points for FLR design and implementation. Developed by William Stephenson in the 1930s, Q methodology combines qualitative and quantitative research techniques to reveal shared subjective viewpoints among stakeholders on a given topic (Tuokuu et al., 2019; Zabala et al., 2018). It is widely used in natural resource governance to analyze stakeholders’ perceptions, beliefs, attitudes, and values, making it particularly relevant for FLR research (Carmenta et al., 2017; Zabala et al., 2018). A Q-study begins with collecting statements (Q-set) relevant to the research topic, typically derived from previous studies, expert consultations, or focus groups(Brown, 1996; Tuokuu et al., 2019). These statements capture diverse perspectives on the issue, facilitating an understanding of both consensus and divergence among stakeholders. Participants then rank and sort these statements on a bell-shaped grid based on their level of agreement or disagreement, with positive rankings on the right and negative rankings on the left (Fig. 1) (Bayala, 2023; Schulze and Matzdorf, 2023; Sumberg et al., 2017). This structured sorting process, known as Q-sorting, results in Q-sorts that represent individual viewpoints. Q methodology enables a nuanced analysis of stakeholder perspectives by identifying areas of agreement and disagreement, which can aid in policy evaluation, conflict mediation, and decision-making (Schulze and Matzdorf, 2023). By revealing underlying patterns in stakeholder perceptions, it offers valuable insights for inclusive FLR governance and participatory policy development.

Subsequent data analysis is conducted using software such as Ken-Q Analysis, PQMethod, and qmethod for R (Sumberg et al., 2017; Zabala et al., 2018), with this study utilizing R software (version R4.1.3). Through this sorting process, the Q methodology integrates quantitative and qualitative data, identifying social viewpoints and attitudinal profiles, which are interpreted through content analysis (Schulze and Matzdorf, 2023).

This study analyses stakeholders’ viewpoints on the key considerations and priorities for designing and implementing FLR in the Tchamba Prefecture of central Togo. It identifies consensus perspectives, prioritizing effective strategies while highlighting factors to avoid. Stakeholders interviewed include local community members, policymakers, and NGOs directly involved in forest and agricultural landscape management. The Q study framework was developed following best practice guidelines and recent literature, particularly Zabala et al. (2018).

### Concourse and Q-set

We established the debate framework by considering the entire spectrum of opinions, drawing from the literature and preliminary field research on stakeholder perspectives regarding implementing FLR. Initially, we brainstormed and crafted statements that reflected various opinions on FLR design. The central question guiding our research was, “How do stakeholders, including local communities, policymakers, extension agents, and NGOs, perceive the FLR design and implementation within the policy process”?

The concourse—representing the full range of ideas, arguments, and perspectives relevant to the central research question—was developed by gathering data from diverse sources. Specifically, we conducted 15 expert interviews, eight focus group discussions (FGDs), and scientific papers relating to FLR (Djenontin et al., 2022) to generate suitable categories for the list of statements included in the research (the Q-set). These categories include Ecological and Conservation Focus, Community and Local Engagement, Governance and Policy-Making, Funding and Incentives and Community Engagement and Social Inclusion. This classification of FLR aspects aligns with frameworks from the literature (Ferretti-Gallon et al., 2022; Löfqvist and Ghazoul, 2019; McLain et al., 2021; Owusu et al., 2021). To enhance the validity of the study design, we held a workshop in Kaboli, Tchamba Prefecture, with representatives from local communities. We tailored the statements to be relevant to FLR. During the final Q-interviews, participants were allowed to suggest changes to the statements if they were discussing a different program. This was done to accommodate varying experiences with the FLR program and to understand how these experiences related to specific program aspects.

Most Q studies recommend including 20–60 statements in a Q-set, depending on the sorting grid’s dimensions (Takase et al., 2024). The statements are ranked in a bell-shaped distribution, allowing for z-score calculations to distill rankings into a single metric (Sumberg et al., 2017). However, smaller Q-sets and participant samples may also be appropriate. Heilbronner et al. (2009), suggests a Q-statement-to-participant ratio of less than 2:1. Given that this study used 24 Q statements, a sample size of around 12 participants aligns with this guideline. However, there is still little evidence to justify any universally recommended sample size, as Kirschbaum et al. (2019) noted. Nonetheless, when formulating opinion statements, we followed the best practice guidelines outlined by Watts and Stenner (2012). They recommend using a balanced and structured Q-set representing multiple opinions and perspectives on the research question. Similarly, following the approach described by Schulze and Matzdorf (2023), statements were carefully framed as affirmatory or negatory to ensure clarity and avoid double negations. We conducted expert and key informant interviews to capture diverse stakeholder perspectives with purposively selected representatives from local communities, NGOs, extension services, and government representatives. Additionally, we organized FGDs across Tchamba Prefecture’s ten cantons, engaging farmers, youth, women, elders, and local leaders. Participants were selected using purposive and convenience sampling, based on their involvement in FLR activities and availability. Tuokuu et al. (2019) emphasize that interviews and FGDs are optimal for capturing a comprehensive range of stakeholder viewpoints.

The Q-set, centred on implementing FLR from stakeholder perspectives, initially consisted of 36 statements. Each participant contributed to a single session, completing one Q-sort. We refined the statement pool through an iterative process to capture stakeholder insights effectively. After removing redundancies and improving clarity, we eliminated 12 statements, resulting in a final selection of 24 statements for analysis (Table 1).

**Table 1:** The Q set—list of statements related to FLR design and implementation, Collated and Reviewed and Finalized for Participant Inclusion

Category	#	Statements	sources
Ecological and Conservation Focus	1	Sustainable forest management practices should be prioritized in the institutional design of forest landscape restoration, even if stricter regulations are required.	(Ewane, 2024)
	2	Emphasising biodiversity conservation should not be a primary goal in the institutional design of FLR projects.	(Pistorius and Freiberg, 2014)
	3	Collaborative research and data-sharing among stakeholders are not essential for informed decision-making in forest landscape restoration.	FGDs/Interviews
	4	Flexibility in restoration approaches is important, allowing adaptation to changing environmental conditions and stakeholder needs.	FGDs/Interviews
	5	Climate change considerations should be integrated into the institutional design of restoration policies to enhance resilience.	(Mansourian et al., 2017a; Tullos et al., 2021)
Community and Local Engagement	6	Incorporating local community input is not essential for successful forest landscape restoration, as it does not ensure alignment with cultural and social values.	(Ewane, 2024), and FGDs
	7	A collaborative approach involving NGOs, local communities, and governments is not the key to achieving comprehensive and successful forest landscape restoration.	(Evans et al., 2023; Schweizer et al., 2021)
	8	Traditional ecological knowledge should be integrated into restoration practices to ensure ecological resilience and sustainability.	(Bayala, 2023)
	9	Stakeholder conflicts over land use and resource allocation can not hinder effective FLR initiatives.	(McLain et al., 2021)
	10	Local empowerment and ownership of restoration projects are vital for long-term success and sustainability.	(Rahman et al., 2023)
Governance and Policy-Making	11	Government-led initiatives are more effective in driving FLR as they provide centralized coordination and resources.	FGDs/Interviews
	12	Balancing economic development and forest restoration goals is challenging but achievable through careful policy-making and stakeholder collaboration	FGDs/Interviews
	13	Economic incentives, such as carbon credits, should be used to incentivise participation in forest landscape restoration.	FGDs/Interview
	14	Adopting a multidisciplinary approach that combines science, policy, and community engagement is not essential for successful restoration.	(Dick et al., 2017)
	15	A tiered approach to policy-making, involving local, regional, and national levels, can lead to more adaptive and contextually relevant restoration strategies.	(Rametsteiner, 2009)
Funding and Incentives	16	Financial incentives and partnerships with private industries are necessary to fund large-scale FLR initiatives.	(Mansourian, 2016)
	17	Private and civil society sector involvement in FLR can lead to innovative solutions and increased efficiency	(Löfqvist and Ghazoul, 2019)
	18	Public awareness and education campaigns do not play a crucial role in gaining support for FLR efforts.	(Ullah et al., 2024)
	19	Funding for restoration should come primarily from public sources to ensure equitable access and distribution.	Interviews
	20	Long-term monitoring and evaluation are critical to assess the effectiveness of FLR initiatives.	(Mills et al., 2023; Vallauri et al., 2005)
Community Engagement and Social Inclusion	21	Improving landownership security while minimizing fragmentation within landholdings can further forest landscape restoration.	(Djenontin et al., 2022)
	22	Women can effectively participate in FLR activities when they have access to resources.	(Djenontin et al., 2022)
	23	International cooperation and agreements are vital to address transboundary challenges in forest landscape restoration	(Mason et al., 2020)
	24	Empowering local communities with decision-making authority does not enhance the success and sustainability of FLR projects.	(Djenontin et al., 2022)

### Case Study

In the framework of the stakeholder mapping of FLR in Tchamba Prefecture, we collected data using an expert interview approach. During these interviews, we discussed the main activities of stakeholders, their connections with each other, their roles in FLR activities, the constraints they face, and their recommendations for improvement. This comprehensive mapping was instrumental in identifying the relevant participants for our study context (the participant set). The main idea was to determine how different stakeholders in FLR perceive various aspects of its implementation. As is standard in Q methodology applications, the primary goal is to identify and synthesize shared perspectives from diverse viewpoints rather than focusing on the representativeness or frequency of these views within a population (Watts and Stenner, 2012; Zabala et al., 2018). This approach enables researchers to uncover the variety of perspectives that exist, irrespective of how prevalent they are among participants. This study involved 15 participants who were purposely selected to capture various opinions on critical issues within the FLR implementation discourse, ensuring a broad representation of viewpoints relevant to the topic.

**Fig. 2:**
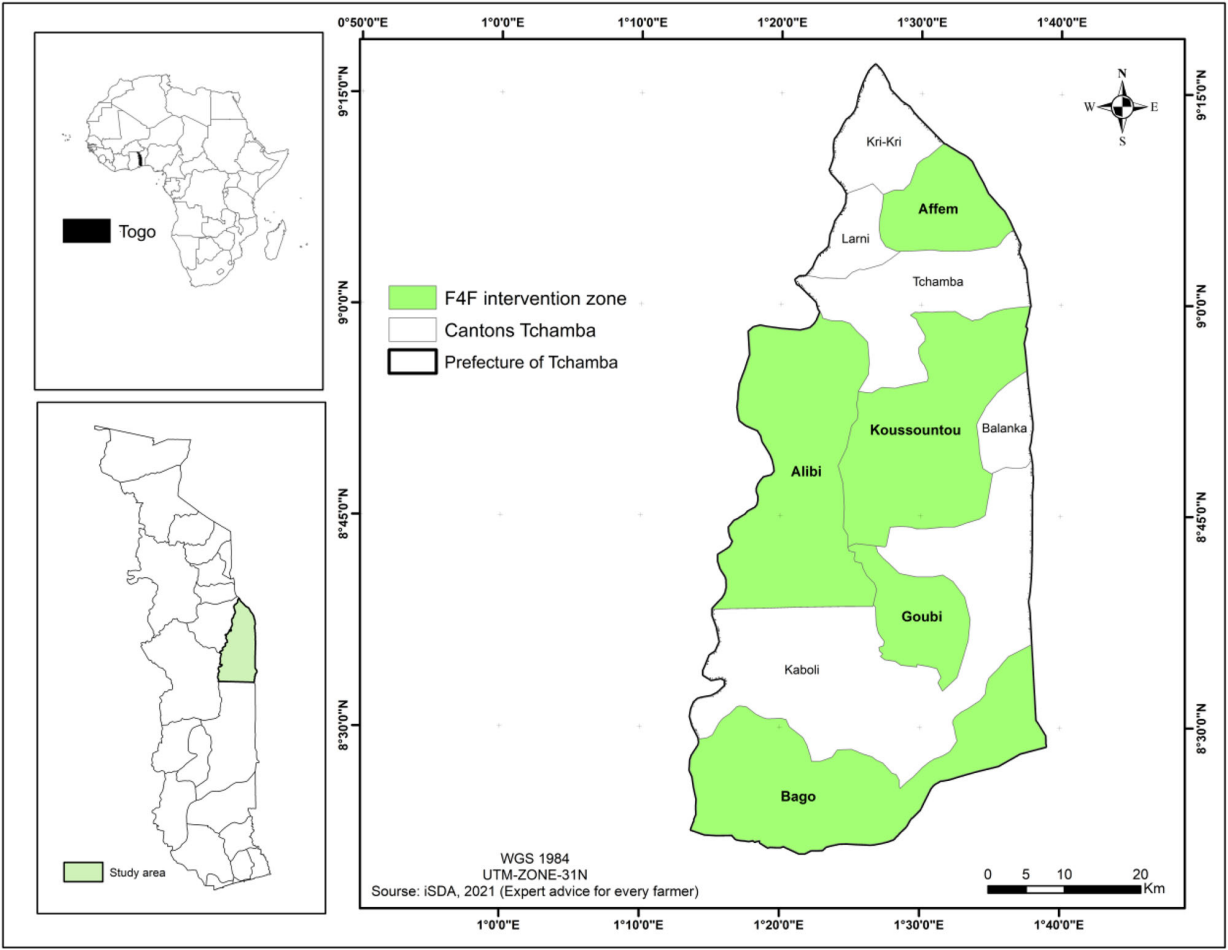
Case study location in Togo: Tchamba Prefecture, Highlighting the Ten Cantons Involved in the Study and the Five Targeted by the F4F Project

We conducted the Q-interviews in the Tchamba Prefecture in central Togo (Fig. 2), involving ten cantons. These are Affem, Alibi, Bago, Balanka, Goubi, Kabolé, Koussountou, Kri-Kri, Larni, and Tchamba. The F4F project primarily focuses on five of these cantons: Affem, Alibi, Bago, Goubi and Koussountou. However, participants were selected from all ten cantons because FLR activities in the five project-focused cantons have broader effects on neighbouring cantons. Additionally, some local initiatives related to FLR have been independently implemented in the other five cantons, making their inclusion relevant for understanding the broader FLR implementation discourse. The participants in the Q-interview represented a range of stakeholders, including members of the local community (across all ten cantons), NGOs, Extension agencies and government representatives from the Ministry of Agriculture, Ministry of Environment and Forestry and the three councils involved in FLR activities under the Global project on FLR and good governance in the forest sector) led by the German Society for International Cooperation (GIZ). The F4F project aims to restore forests and tree-rich, fertile landscapes in selected countries, including Benin, Cameroon, Ethiopia, Madagascar, and Togo. It seeks to improve governance in the forest sector, combat climate change, and enhance biodiversity and water availability by restoring degraded lands. Additionally, the project aims to increase the incomes of households managing forest landscapes and agroforestry systems, focusing on supporting women and young people (GIZ, 2023).

Tchamba Prefecture is located in a tropical semi-humid climate zone of the Sudano-Guinean type, characterized by a monomodal rainfall pattern. This pattern includes a long rainy season from April to October, followed by a short dry season from November to March (Adjonou et al., 2010). The Prefecture features vegetation extending from Upper Côte d’Ivoire’s savannah forests. The region experiences a single rainy season, with average annual rainfall between 1200 and 1300 mm.

### Participant Set

As FLR programs are mainly implemented locally, it is important to take into account the stakeholders at the appropriate level. In the accompanying research project TREES (Tropical Restoration Expansion for Ecosystem Services) framework, we investigate recommendations for the global F4F project (country package Togo). For this purpose, We started the TREES project financed by the German Federal Ministry for Economic Cooperation and Development (BMZ), commissioned and administered through F4F of the Deutsche Gesellschaft für Internationale Zusammenarbeit (GIZ), in June with stakeholder mapping. During this initial phase, we accessed a range of relevant actors who were interested in the project.

#### Local communities

Local communities are key stakeholders in this study, as they own land in Togo. It is their decision whether to allocate their land for restoration purposes, and their involvement is crucial for the success of any FLR initiatives in the country. Local communities play an important role in the conservation of natural resources management (Kokou et al., 2008). Additionally, engaging local communities in the planning and implementation of FLR projects can help ensure that their needs and priorities are met, leading to more sustainable outcomes (Ewane, 2024). In Togo, local communities are represented by the cantonal development committee (CCD) and the village development committee (CVD), whose perspectives on FLR implementation design are essential to understand. Therefore, engaging these committees to gain insights into local perspectives and ensure effective and inclusive FLR implementation is crucial.

#### International and local NGOs

NGOs are also important in this research. They play an important role in tackling environmental cgallenges, such as, land and forest degradation, particularly in regions like Tchamba. These organizations often operate at the grassroots level, implementing programs that directly engage local communities in sustainable practices and conservation efforts (Nikkhah and Redzuan, 2010). In forestry, NGOs contribute by promoting reforestation and afforestation initiatives, advocating for sustainable forest management, and raising awareness about the impacts of deforestation. They also help mitigate forest degradation through various restoration projects, including tree planting and the development of agroforestry systems. Specifically, in Tchamba, NGOs such as AGAIB and AE2D are actively involved in environmental conservation and FLR activities. AE2D focuses on tree planting and environmental sustainability, while AGAIB works on climate change mitigation through carbon credit projects. Other NGOs, including AJA, APCR, and ADCF, contribute to various aspects of FLR in the region, enhancing collaborative efforts toward environmental sustainability and restoration. These NGOs provide vital support by mobilizing resources, offering technical expertise, and facilitating community participation in FLR efforts. Their work helps restore degraded landscapes, enhances local livelihoods, and contributes to the overall resilience of ecosystems. Through their dedicated efforts, NGOs in Tchamba are instrumental in promoting sustainable development and achieving long-term environmental goals.

#### Government representatives and policymakers

This stakeholder group is divided into two subgroups: the centralised and decentralised local authorities. The centralised authorities we interviewed include the Ministry of the Environment and Forest Resources (MERF) regional representatives and the Ministry of Agriculture, Livestock, and Rural Development (MAEDR). The decentralised local authorities, particularly the three councils of the prefecture (Tchamba I, Tchamba II, and Tchamba III), are increasingly involved in FLR initiatives through direct action with the local population or collaboration with the prefecture’s NGOs. These actions include intensifying communication-related to FLR and their consideration in the councils’ management plans. They serve as policymakers.

#### The extension agents

Extension agents are essential for the success of FLR efforts in Togo. They are employed by state own organisations such as the Togo Institute for Technical Advice and Support (ICAT), the Office for the Development and Exploitation of Forests (ODEF), and the National Agency for Grassroots Development Support (ANADEB). These agents bring valuable expertise from their backgrounds in agricultural sciences and forestry. They provide technical guidance, facilitate the implementation of sustainable practices, and support local communities in adopting restoration activities (Ullah et al., 2024). Extension agents play a crucial role in connecting scientific information with practical implementation. By doing so they can ensure that FLR initiatives are effectively tailored to the unique ecological and socio-economic conditions of the regions they serve. Moreover, it is crucial to consider their perspectives, as their on-the-ground experience and insights can significantly influence the design and execution of restoration projects.

### Interviews

In Q methodology research, the diversity of respondents is more important than the sample size (Sumberg et al., 2017). Significant results can be obtained even with small samples (Zabala et al., 2018). In fact, in some cases, the robustness of perspective groupings does not strongly depend on the number of respondents (Watts and Stenner, 2005). Additionally, the selection of participants for a Q study is generally targeted (Zabala et al., 2018). Although the Q methodology is flexible, it involves the ranking of statements, which requires strong reading and writing skills (Bayala, 2023). For this study, we conducted face-to-face interviews in August-September 2023. The participant set consisted of 15 people: extension agents (2), local community group representatives (3), NGO leaders (5), and government representatives (policymakers) (5). For this study, proficiency in French was an important selection criterion.

**Fig. 3:**
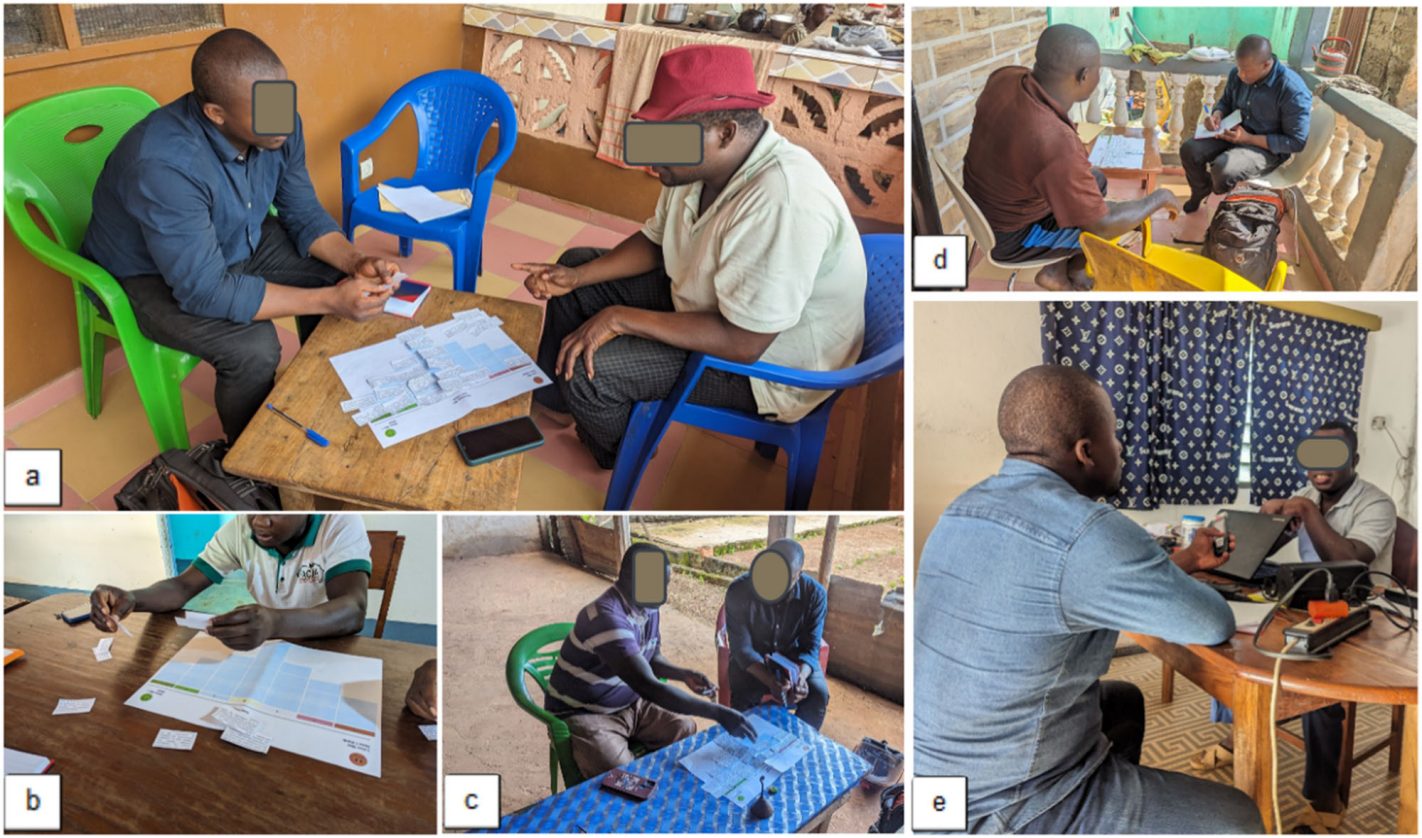
Participants sorting the statements (**a**–**c**) and reporting interviews (**d**, **e**). Courtesy: authors, 2023. Source: Field data, 2023

After identifying the participants, we arranged individual appointments with each of them to conduct the Q-set. We explained the purpose of the study and obtained informed consent from the respondents before asking them to sort the statements they received into card form. When contacting the interviewees, we emphasised that the interview aimed to explore their personal views on the design and implementation of FLR in the Tchamba Prefectureand that the sorting of the statements was not a test of their knowledge. Interviews were hand-reported and recorded only with the interviewee’s consent (Fig. 3d, e). First, the statements were read aloud and handed to the participants. Before beginning the actual sorting, participants created piles of statements categorized as “agree,” “neutral,” and “disagree.” During this process, participants were encouraged to provide explanations for their initial reasoning.

A grid consisting of a 7-point scale ranging from −3 (‘less like how I think’) to +3 (‘more like how I think’) was applied (Fig. 1). In the second phase, participants were instructed to fill in the left and right sides of the grid (Fig. 3a–c) using a scale from −3 to +3, prior to completing the center of the grid. We documented the changes between statements perceived as positive, neutral, and negative, which allowed us to more precisely identify where participants’ feelings turned from positive to negative (Watts and Stenner, 2012). Once sorted, we asked participants to clarify the meaning of the statements placed at the ends of the grid (+/−3 and +/−2 columns), as these represented the most polarised opinions of the interviewees.

### Data Analysis

We used the freely accessible ‘qmethod’ package (version 1.8) for R proposed by Zabala (2014) to analyze the data collected from the interviews. Initially, the data from the Q-sets administration was entered into Excel files, with respondents as rows and statements as columns. These Excel files were then converted into CSV files before being imported into R for further analysis. The Q sorts were intercorrelated using Pearson correlation. Following this, principal component analysis (PCA) was employed to reduce the dimensionality of the dataset by identifying latent components based on the previously calculated correlations. After conducting the PCA, we applied varimax rotation, an orthogonal rotation technique, to maximize the variance of each perspective loading. Other rotation techniques also returned three perspectives with similar Q-sort allocations. However, after considering the different statistical criteria behind these techniques, we concluded that varimax rotation was the most appropriate for our study. Extracting perspectives involves condensing all individual responses (Q-sorts) into a few distinct groupings, each representing a shared viewpoint among respondents with similar perspectives (Zabala et al., 2018). The number of perspectives was determined using the following statistical criteria:

- The Kaiser-Guttman criterion suggests retaining perspectives with an Eigenvalue of 1 or higher (Guttman, 1954)
- Humphrey’s Rule recommends selecting perspectives with cross products of the two highest loadings that exceed twice the standard error (Watts and Stenner, 2012)
- Perspectives that have two or more significant loadings after extraction (Watts and Stenner, 2012)

A preliminary interpretation of perspectives, considering their realism and similarities, helps guide the selection of perspectives for analysis (Zabala et al., 2018). Typically, studies analyse three to five perspectives. The first perspectives capture most variability in the correlation matrix, making them essential for a clearer data structure (Buckwell et al., 2020; Zabala, 2014). Comparing different perspective combinations is also advisable (Bayala, 2023). Therefore, we initially rotated five perspectives, then four, and finally three. These criteria retained three perspectives for our Q set (P1, P2, P3). Perspective correlations highlighted distinctive and consensual statements (Appendices Table A1), and perspective loadings provided insights into the correlation between perspectives and respondents (Table 4). Following Schober et al. (2018), We used a conservative score of 0.7 or higher to identify significant components, ensuring stronger statistical correlations (Bayala, 2023).

Table 2 summarises the three-perspective solution’s key properties according to the earlier criteria. Consequently, each perspective encompasses at least two Q-sorts, has an Eigenvalue greater than one, and collectively, the perspectives account for more than 30 percent of the variance in the data.

**Table 2:** General characteristics of the Three-perspective Solution, Including Eigenvalues, Number of Q-Sorts Per Perspective, and Variance Explained

Perspectives	Number of Q-sorts	Eigenvalue	Variance explained (in %)
1	7	4.32	28.82
2	6	4.21	28.04
3	2	2.59	17.27

In summary, each perspective represents a distinct viewpoint component on the design of FLR implementation, which is reflected in a Q sort typical of how participants loading on that perspective responded. The statistical process behind these typical Q sorts involves calculating z-scores for each statement- perspective combination, indicating the degree to which a statement contributes to a perspective. By examining the differences in z-scores across perspectives, we can identify the statistical distinctions among perspectives and pinpoint conflict and consensus statements among the various viewpoints (Schulze and Matzdorf, 2023). Analyzing the dispersion of statement z-scores (Appendice: Figure A) for each perspective allows us to determine if a perspective significantly distinguishes a statement, which is captured by calculating the standard error of the differences in respective z-scores for a statement.

## Results

The qualitative aspect of the analysis involves interpreting the various perspectives to construct the study’s viewpoints. We compared the statements that elicited the most divergent opinions and subsequently analyzed the distinguishing statements for each perspective (Appendices Table A and Table B). Additionally, we incorporated interview information to provide context and further interpret the viewpoints.

### Consensus

Before tackling the divergent issues related to the institutional design of FLR, it is essential to identify areas of stakeholder consensus. All participants agreed that biodiversity conservation should be a primary goal in the institutional design of FLR projects (Statement 2, hereafter referred to as ST2). Flexibility in restoration approaches was widely supported (ST4), emphasizing adaptation to environmental and stakeholder needs. Similarly, integrating climate change considerations into FLR policies was seen as crucial (ST5). Regarding land use, participants concurred that Stakeholder conflicts over land use and resource allocation could hinder effective FLR initiatives (ST9).

Stakeholders widely agreed on key governance aspects of FLR. Economic incentives, such as carbon credits, were seen as essential for participation in FLR (ST13). A tiered policy-making approach across governance levels was favored for adaptability (ST15). Financial incentives and partnerships with private industries were recognized as necessary to fund large-scale FLR initiatives (ST16). The involvement of the private and civil society sectors in FLR was believed to lead to innovative solutions and increased efficiency (ST17). Furthermore, public awareness and education campaigns were highlighted as vital in gaining support for FLR efforts (ST18). Stakeholders also agreed that women can effectively participate in FLR activities with access to resources (ST22). Finally, there was a consensus on the importance of empowering local communities with decision-making authority to enhance the success and sustainability of FLR projects (ST24).

### Interpretation of Perspectives related to FLR design and implementation in Tchamba Prefecture

Table 3 presents the three perspectives derived from the statements related to our research question. The interpretation of the three perspectives outlined in Table 3 is as follows.

**Table 3:** Interpretation of Statements and Their Associated Perspectives on the Implementation Design of FLR in Tchamba Prefecture

Statement Number	Perspectives		
	P1	P2	P3
1	2	0	−3
2	−1	−2	−1
3	−1	−3	−2
4	1	0	1
5	0	0	1
6	−3	−1	−3
7	−2	−2	1
8	3	1	1
9	−3	−1	−2
10	0	3	0
11	−1	0	0
12	2	0	1
13	2	2	3
14	−1	−3	3
15	1	1	0
16	3	2	2
17	1	1	0
18	−2	−2	−1
19	−2	0	−1
20	0	3	0
21	1	2	2
22	0	1	0
23	0	−1	−1
24	0	−1	−2

#### Perspectives 1—Incentive-Driven Restoration

According to our respondents, offering financial incentives, such as carbon credits or partnerships with private industries, is considered the most important driver of FLR success. The first perspective accounts for 28.82% of the study variance, and seven respondents are loaded into it. Participants loading onto this perspective share similar viewpoints, as reflected in their Q-sorts. A high loading indicates that a participant’s responses closely align with the shared perspective. Regarding the stakeholder group affiliation, two people belong to the local communities: one from NGOs, two from extension services, and two from policymakers. This perspective represents a viewpoint that emphasizes the integration of economic and environmental goals in FLR (ST1, +2; ST12, +2; ST13, +2; ST16, +3). It underscores the importance of using financial incentives and fostering partnerships with private industries to motivate participation and secure funding. This perspective advocates for a strategic and collaborative approach, where careful policy-making and stakeholder collaboration are essential to balancing economic development and environmental sustainability.

The +3 score for statement 16 reflects strong agreement that financial incentives and private-sector partnerships are crucial for FLR implementation. This highlights their role in funding large-scale restoration efforts, justifying the “Incentive-Driven Restoration” label. Respondent 1 stated, *“For the successful implementation of FLR, incentives are necessary due to the ineffectiveness of public authorities. Currently,NGOs are primarily responsible for conducting restoration activities in our locality”*. Respondent 4 further stated*, “Involving private companies through contractual agreements that provide funding for reforestation can be effective. Even if the funder harvests the wood after five years, the credit provided will still ensure the success of the reforestation initiative.”*. Additionally, the +3 score for Statement 8 indicates strong agreement on the importance of traditional ecological knowledge (TEK) for FLR success. Respondents emphasized that local and indigenous practices enhance ecological resilience, sustainability, and cultural relevance in restoration efforts. Integrating TEK ensures that FLR initiatives are scientifically sound, culturally respectful, and sustainable in the long term.

Furthermore, the score of -3 for the statements suggesting that local community input is not essential and that stakeholder conflicts over land use do not hinder FLR indicates strong disagreement among respondents. This consensus reflects the belief that successful FLR initiatives must incorporate local community input to align with cultural and social values. Additionally, respondents believe that resolving stakeholder conflicts over land use and resource allocation is critical, as such conflicts can significantly hinder the effectiveness of FLR efforts (ST9, -3). This underscores the importance of community engagement and conflict resolution in implementing successful FLR projects. Respondent 2 stated, *“Involving the local community is essential for any project within their area, as their participation is crucial for success. Without their involvement, the local population is unlikely to contribute. Furthermore, unresolved land issues can impede the initiation of restoration initiatives”*.

In summary, regarding what should and should not be considered in the implementation design of FLR, Perspective 1 emphasizes integrating economic and environmental goals in FLR. Key strategies include using financial incentives, such as carbon credits, and fostering partnerships with private industries to secure funding and motivate participation. Respondents agree on the necessity of these financial mechanisms for successful FLR implementation. Additionally, there is strong support for incorporating traditional knowledge to enhance ecological resilience and sustainability. This Perspective also underscores the importance of community engagement and conflict resolution over land use and resource allocation to ensure that FLR initiatives are culturally aligned and effective.

#### Perspectives 2—Comprehensive and Collaborative Restoration

Perspectives 2, highlights the significance of taking a holistic, inclusive, and multidisciplinary approach to FLR. It stresses the importance of empowering local communities, implementing long-term monitoring, and involving a range of stakeholders, such as NGOs, local communities, and governments, to achieve successful restoration outcomes. The second perspective accounts for 28.04 per cent of the study variance, and six respondents loaded into it. Regarding the stakeholder group affiliation, one person belongs to the local communities, three are from NGOs, and two are policymakers.

Respondents emphasize the integration of biodiversity conservation (ST2, -2), collaborative research and data-sharing among stakeholders (ST3, -3), viewing these elements as critical for informed decision-making and successful restoration efforts. Respondent 3 stated, “*The role of biodiversity, especially in soil conservation, is crucial for restoring soil health. Contemporary agricultural practices differ greatly from traditional methods historically focused on preserving soil fertility. In the past, extensive farming techniques were common, but modern practices often involve continuous cultivation of the same areas for multiple years, resulting in soil degradation. Additionally, the widespread use of chemical inputs in modern agriculture poses a significant threat to soil microorganism populations, worsening soil health deterioration”*.

There is strong agreement on the importance of local community empowerment and ownership (ST10, +3), long-term monitoring and evaluation (ST20, +3), and the use of economic incentives like carbon credits (ST13, +2). Similarly to perspective 1, financial incentives and partnerships with private industries (ST16, +2) are crucial for funding large-scale initiatives. Like in perspective 1, respondents also moderately disagree with ST 18 (-2), indicating a similar recognition of the importance of public awareness and education campaigns in gaining support for FLR efforts. This perspective aligns with the holistic and collaborative approach emphasized in Perspective 2, where stakeholders emphasize the necessity of comprehensive strategies incorporating public engagement and awareness to ensure successful restoration outcomes. Improving landownership security while minimizing fragmentation (ST21, +2) is also essential. Neutrality regarding the statements ST1, ST4, ST5, ST11, ST12, and ST19 indicates a lack of strong consensus among respondents (loaded in perspective 2) regarding those particular aspects of FLR. While some statements may evoke explicit agreement or disagreement, neutral responses suggest that respondents may have varied perspectives or may not consider those aspects to be as critical or impactful in the context of FLR.

This perspective highlights that successful FLR requires a comprehensive strategy incorporating scientific, policy, and community engagement to ensure ecological resilience, sustainability, and broad-based support.

#### Perspectives 3—Incentive-Driven Community Restoration

Participants loading on this perspective emphasized that economic incentives, such as carbon credits, should be used to incentivise participation in FLR (ST13, +3). The last perspectives accounts for 17.27% of the study variance, and two respondents loaded into it. Regarding the stakeholder group affiliation, one person is from the NGOs, and the other is from the extension services. Similarly, *“Many individuals are attracted to farming because of the potential for annual income generation from crops like maize or soybeans. However, reforestation efforts are often met with skepticism and ridicule. To address this, incentivizing reforestation through economic mechanisms such as carbon credits could greatly enhance motivation and participation. Suppose individuals are guaranteed to receive an income from reforestation efforts at the end of each year. In that case, their involvement in such activities is likely to increase, ultimately leading to a significant expansion of forest cover in our region”* (Resp4). The score of +3 for the statement “Adopting a multidisciplinary approach that combines science, policy, and community engagement is not essential for successful restoration” indicates strong agreement among respondents within Perspective 3. This suggests that respondents in this Perspective believe that a multidisciplinary approach, which integrates science, policy, and community engagement, is not crucial for FLR success. Instead, they may prioritize other approaches or factors that are more critical to the success of FLR initiatives. This perspective implies a potential emphasis on more focused or specialized strategies, possibly valuing specific expertise or direct interventions over broader, integrated efforts involving multiple disciplines and stakeholder engagement.

Similar to the argument stressed by respondents loaded in Perspective 1 and Perspective 2, respondents in this Perspective view financial incentives and partnerships with private industries (ST16, +2) as crucial for funding large-scale initiatives. Respondents within perspective 3, like those loaded in perspective 2, also view improving landownership security while minimizing fragmentation (ST21, +2) as essential for FLR. The score of -3 for the statement “Sustainable forest management practices should be prioritized in the institutional design of landscape restoration, even if it requires stricter regulations” indicates strong disagreement among respondents within Perspective 3. This suggests that respondents in this perspective do not believe that prioritizing sustainable forest management practices is necessary if it involves implementing stricter regulations. They may perceive such regulations as potentially burdensome, counterproductive, or not essential for achieving effective landscape restoration. This strong disagreement implies a preference for more flexible, less regulated approaches to forest landscape restoration, potentially emphasizing other methods or strategies that do not require stringent regulatory frameworks. *“It is widely recognized that vegetation cover is diminishing, and current data indicates that only 24.24% of our forest resources remain due to ongoing deforestation. Since the last forest inventory conducted by GIZ, the current status of forest resources remains uncertain. I believe tree planting should be a cultural practice rather than one strictly governed by regulations. It should be driven by an awareness of the importance of nature and a commitment to future generations”* Resp9.

Similar to the argument stressed by respondents loaded in Perspective 1, the score of -3 for the statement “Incorporating local community input is not essential for successful forest landscape restoration, as it does not ensure alignment with cultural and social values” indicates a strong disagreement among respondents within this Perspective. This reflects a consensus that successful FLR initiatives must incorporate local community input to align with cultural and social values. Respondents in this Perspective emphasise the collaborative research and data-sharing among stakeholders (ST3, -2), viewing this element as critical for informed decision-making and successful restoration efforts. Respondents believe that resolving stakeholder conflicts over land use and resource allocation is critical, as such conflicts can significantly hinder the effectiveness of FLR efforts (ST9, -2). Respondents further disagree with the statement, ‘Empowering local communities with decision-making authority does not enhance the success and sustainability of FLR projects’ (ST24,-2). This indicates that they believe empowering local communities with decision-making authority is important for the success and sustainability of FLR projects. Their disagreement suggests that involving local communities in decision-making is a positive and necessary factor for effective restoration efforts.

### Statements of complete disagreement

The statement “Sustainable forest management practices should be prioritized in the institutional design of landscape restoration, even if it requires stricter regulations” receives varying degrees of agreement among respondents across different perspectives. In perspective 1, there is substantial agreement ( + 2) with prioritizing sustainable forest management practices, indicating that respondents in this group believe it should be a central focus of institutional design, even if it entails implementing stricter regulations. However, in perspective 2, there is neutrality (0), suggesting that respondents in this group neither strongly agree nor disagree with the statement. Conversely, in perspective 3, there is very strong disagreement (-3) with prioritizing sustainable forest management practices with stricter regulations, indicating that respondents in this group do not believe such regulations are necessary or desirable for effective landscape restoration. This divergence in perspectives highlights respondents’ complexity and differing priorities regarding integrating sustainable practices and regulatory measures in landscape restoration efforts.

### Correlations Between Perspectives and Respondents

The perspective loadings presented in Table 4 illustrate how participants’ views correlate with each perspective. Correlation coefficients range from −1 to +1, where 0 denotes a negligible correlation (Schober et al., 2018). Higher perspective loading scores, approaching −1 or 1, signify a stronger correlation between participants and the corresponding perspectives, with negative scores indicating disagreement and positive scores indicating agreement (Bayala, 2023). These loadings facilitate the identification of conflicting and shared perspectives, as well as discourse alliances (O’Riordan et al., 2019)

**Table 4:** Q-sort perspective loadings

Respondents	Perspectives		
	P1	P2	P3
Resp1 (local community representative/CVD)	0.63	0.53	−0.077
Resp2 (local community representative/CVD)	0.78	0.22	0.080
Resp3 (Extension services/ANADEB)	0.82	0.26	0.143
Resp4 (local community representative/CCD)	0.24	0.81	0.158
Resp5 (NGO engaged in environment/AJA)	0.47	0.64	0.293
Resp6 Centralised authority/Regional representative for MERF	0.68	0.26	0.472
Resp7 (NGO engaged in forest ecosystems conservation/ADCF)	0.18	0.82	0.239
Resp8 (NGO promoting carbon credit/AGAIB)	0.72	0.24	0.428
Resp9 (NGO promoting carbon credit/AGAIB)	0.23	0.17	0.868
Resp10 (Decentralised local authorities/Mayor of Tchamba1.)	0.41	0.75	0.011
Resp11(NGO engaged in trees planting/AE2D)	0.30	0.71	0.406
Resp12(Extension services/ICAT)	0.69	0.35	0.326
Resp13 (Decentralised local authorities/Mayor Tchamba 2.)	0.67	0.40	0.158
Resp14 (Decentralised local authorities/Mayor Tchamba 3.)	0.34	0.72	0.324
Resp15 Centralised authority/Regional representative for MAEDR	0.13	0.25	0.910

*Resp* respondent

In the context of FLR design and implementation, the perspective loadings underscore the influence of the Incentive-Driven Restoration perspective. Perspective 1 highlights the pivotal role of incentives, such as financial mechanisms and partnerships with private industries, in motivating participation and securing funding for FLR initiatives. The dominant scores within this perspective reflect a consensus among stakeholders, including representatives from the local community (Resp2: 0.78), extension services (Resp3: 0.82), and NGOs promoting carbon credits (Resp8: 0.72), indicating strong alignment with the principles of incentive-driven restoration. This unanimity suggests a collective endorsement of strategies emphasizing economic incentives to drive successful FLR implementation.

In Comprehensive and Collaborative Restoration (perspective 2), the perspective loadings illustrate a unified stance among diverse stakeholders towards holistic FLR approaches. Dominated by representatives from various sectors, including local communities (Resp4: 0.81), NGOs engaged in forest ecosystems conservation (Resp7: 0.82), Decentralized local authorities (Mayor of Tchamba1: 0.75, Mayor Tchamba 3: 0.72), and an NGO focused on trees planting (Resp11: 0.71), this perspective signifies broad-based support for inclusive and multidisciplinary restoration strategies. The absence of conflicting views underscores a shared vision among actors, emphasizing the importance of community empowerment, long-term monitoring, stakeholder collaboration, and the integration of biodiversity conservation. This alignment underscores a collective commitment to comprehensive and collaborative approaches in the institutional design of FLR initiatives.

In the domain of Incentive-Driven Community Restoration (perspective 3), perspective loadings indicate a convergence of perspectives primarily led by two key stakeholders. Dominated by the views of the NGO promoting carbon credit (Resp9: 0.868) and the Centralized authority (Regional representative for MAEDR: 0.91), perspective 3 highlights a focused alignment on leveraging economic incentives to drive community engagement in restoration efforts. The absence of conflicting views across diverse actors underscores a unified stance toward incentivizing participation through mechanisms such as carbon credits. This unity suggests a shared recognition of the importance of economic incentives and centralized authority in promoting community-driven restoration initiatives within the FLR framework.

## Discussion

The results of this study show that four different stakeholder groups coexist when designing and implementing FLR initiatives: local community representatives, NGOs engaged in environmental conservation and restoration, Extension Services, and government representatives. Despite being influenced by the same landscape dynamics, these groups perceive and prioritize strategies differently, with perceptions shaped by personal experiences and individual interests. This leads them to emphasize different strategies for forest landscape restoration, where what some stakeholders view as a priority strategy may be seen as less critical by others. Importantly, our results indicate that different viewpoints do not strictly correspond to the different stakeholder groups, and thus, a mismatch among stakeholder groups poses no challenge to the development and implementation of FLR. Instead, it is possible to derive three strategies and approaches essential for successful FLR design.

The investigations of these various perspectives have allowed the identification of consensual understandings among FLR stakeholders that could form a basis for developing integrated and effective FLR policies and strategies in Togo. It will be easier to engage stakeholders about strategies they already agree on. According to Carmenta et al. (2017), Stakeholders will only participate in a process if they perceive it to be advantageous to them. Consensus serves as the foundation for collaborative efforts and multi-stakeholder processes needed for implementing a landscape approach (Sayer et al., 2013). In northern Ghana, implementing a landscape approach required seeking a consensus on problems and challenges affecting the landscape and its governance, constituting the common concern entry point (Bayala, 2023). In this study, we argue that directly incorporating consensus attitudes identified through the Q-methodology into policy development is a critical step for the effective design of FLR.

Despite differing perspectives, stakeholders reached a consensus on key issues critical to FLR success, ensuring ecological sustainability and stakeholder engagement. All participants agreed on the importance of biodiversity conservation, recognizing its role in ecosystem resilience. There was also unanimous support for flexibility in restoration approaches, allowing adaptation to changing environmental conditions and stakeholder needs. Climate change considerations were universally seen as essential in FLR policy design, emphasizing the need for climate resilience. Additionally, stakeholders highlighted the importance of conflict resolution in land use and resource allocation to improve FLR effectiveness. Economic incentives, such as carbon credits, were widely supported, reflecting a shared belief in financial mechanisms to motivate participation and secure funding.

While stakeholders agreed on several issues, significant differences also emerged. Economic incentives, such as carbon credits and private-sector partnerships, were seen as key motivators for FLR participation. This aligns with research showing that financial incentives promote sustainable land management (Akita and Ohe, 2021). Similarly, Ewane (2024) highlights their role in enhancing community participation in restoration efforts. Our findings also emphasize the integration of traditional ecological knowledge in FLR, supported by Reyes-García et al. (2019), who argue that local and indigenous knowledge strengthens ecological resilience.The analysis results of perspective 2 underscores the necessity of a holistic, inclusive, and multidisciplinary approach to FLR. This perspective is supported by research advocating for integrated landscape approaches that consider biodiversity conservation, soil health, and ecosystem services (Reed et al., 2016). Rahman et al. (2023); Ullah (2024) further emphasised the significance of empowering local communities and ensuring their active involvement in FLR projects. They highlight that community engagement and ownership are crucial for restoration efforts’ long-term success and sustainability.

Perspective 3 highlights the role of economic incentives in driving community engagement in FLR. This aligns with Hounkpati et al. (2024b), who argue that financial returns from restoration activities significantly enhance local participation and commitment. Our study also reveals a preference for flexible, less regulated approaches, contrasting with studies advocating for stricter regulatory frameworks to ensure compliance and effectiveness in natural resource conservation (Jiang and Jiang, 2023). This divergence underscores the importance of tailoring FLR policies to local contexts and stakeholder preferences. Research on FLR in Indonesia supports this flexible governance approach, emphasizing stakeholder mobilization, conflict management, and decision-making at the landscape level (Van Oosten et al., 2014). Instead of rigid design criteria, these cases illustrate how public-private institutional arrangements can enhance restoration efforts. In Tchamba Prefecture, this flexibility allows for adaptive strategies, aligning governance with local conditions and stakeholder needs, ultimately fostering more effective and sustainable FLR outcomes.

Comparing our findings with FLR initiatives in other regions reveals commonalities and differences. Like our study, Mahar and Schneider (2023) found that economic incentives and community empowerment were key to successful FLR in Latin America. However, Choi et al. (2019) emphasize government-led initiatives and strict regulations as critical in East Asia. These regional differences suggest that while economic incentives and community engagement are universally relevant, FLR strategies must be adapted to local socio-political and ecological contexts (Djenontin et al., 2018). The policy recommendations derived from this study underscore the need for integrating economic and environmental goals, promoting inclusive and multidisciplinary approaches, and ensuring community-driven restoration practices. These recommendations align with global best practices and guidelines from international organizations such as the International Union for Conservation of Nature (IUCN) and the World Resources Institute (WRI). Implementing these policies in Togo can enhance the effectiveness and sustainability of FLR initiatives, contributing to broader environmental and socio-economic benefits.

While this study provides valuable insights into stakeholder perspectives on FLR, it is important to acknowledge the limitations of our research. One key limitation is the relatively small sample size of 15 respondents. Although Q-methodology does not require large samples for statistical generalization and instead focuses on capturing a diversity of viewpoints, we recognize that a larger sample size could have strengthened the representation of perspectives, particularly among local communities. However, there is little evidence to justify the sample size recommendation (Churruca et al., 2021; Kirschbaum et al., 2019). Future research should increase the sample size to improve representativeness and validate findings across diverse stakeholder groups. Expanding the sample will also contribute to more comprehensive policy recommendations. Additionally, studies should explore the long-term impacts of FLR strategies on ecological resilience and community livelihoods. Comparative studies across different ecological and socio-economic settings can provide deeper insights into the contextual factors that influence the success of FLR initiatives. Investigating the role of innovative financing mechanisms and technologies in scaling up FLR efforts can further inform policy and practice, ensuring more effective and sustainable restoration outcomes.

## Conclusion

We investigate the stakeholder viewpoints on the FLR design and implementation in the Tchamba Prefecture of Togo. The study identifies four major groups of stakeholders: the local community, NGOs (engaged in resource conservation and ecological restoration), extension services, and government representatives. This plurality of stakeholders implies a complexity and diversity of perspectives on FLR design in the Tchamba Prefecture. The institutional design of FLR initiatives needs to take various forms to accommodate the diverse preferences of these individual stakeholders. Our findings provide valuable insights for developing effective and inclusive restoration policies by identifying key perspectives and areas of consensus and divergence. Specifically, economic incentives, community engagement, and flexible governance approaches emerge as critical factors for FLR success. Integrating economic, environmental, and social dimensions in FLR strategies is crucial for achieving sustainable and resilient landscapes. Additionally, our study underscores the importance of tailoring FLR policies to local contexts and stakeholder preferences, suggesting that flexible, less regulated approaches can enhance restoration efforts. Methodologically, this study highlights the value of mixed-methods approaches in capturing the multifaceted nature of stakeholder perspectives and the dynamic processes of FLR. Future research should continue exploring stakeholder engagement dynamics and the impacts of different restoration approaches. Comparative studies and innovative financing mechanisms will be valuable for scaling up FLR efforts globally.

## Supplementary information

Appendices_QMETHOD_FLR

## Data Availability

The datasets generated during and/or analysed during the current study are available from the corresponding author upon reasonable request.
