# Supplementary material for: Institutional Design of Forest Landscape Restoration in Central Togo: Informing Policy-making through Q Methodology Analysis
Source: Environ Manage. 2025 Mar 3;75(5):1239–56. doi: 10.1007/s00267-025-02137-x (PMC12033111; doi:10.1007/s00267-025-02137-x)
Supplement: Supplementary file 1 — Appendices_QMETHOD_FLR [file 267_2025_2137_MOESM1_ESM.zip › Appendices_QMETHOD_FLR.docx]

**Appendices**

**Institutional Design of Forest Landscape Restoration in Central Togo: Informing Policy-making through Q Methodology Analysis**

**Environmental Management.**

Hamza Moluh Njoya^1, 2, 3^ [^ORCID^](https://orcid.org/0000-0002-5067-2871) ^*^, Kossi Hounkpati^1, 4^ [^ORCID^](https://orcid.org/0000-0003-0212-7331), Kossi Adjonou ^4^ [^ORCID^](https://orcid.org/0000-0001-8491-8107), Kouami Kokou^4^ [^ORCID^](https://orcid.org/0000-0002-2400-0852), Stefan Sieber^1, 2^ [^ORCID^](https://orcid.org/0000-0002-4849-7277) , Katharina Löhr^1, 5^ [^ORCID^](https://orcid.org/0000-0003-2691-9712)

*^1^Leibniz Centre for Agricultural Landscape Research (ZALF), Sustainable Land Use in Developing Countries, 15374 Müncheberg, Germany*

^2^ *Department of Agricultural Economics,* *Faculty of Life Sciences, Humboldt Universität zu Berlin, 10099 Berlin, Germany*

*^3^ Department of Rural Socio-Economics and Agricultural Extension, Faculty of Agronomy and Agricultural Sciences of the University of Dschang, 222 Dschang, Cameroon.*

*^4^ Forest Research Laboratory, Climate Change Research Centre, University of Lomé, 1515 Lome, Togo*

*^5^ Urban Plant Ecophysiology, Humboldt Universität zu Berlin, 10099 Berlin, Germany*

^*^Corresponding author: [*Hamza.Moluh-Njoya@zalf.de*](mailto:Hamza.Moluh-Njoya@zalf.de)

Table A: Flagged Q-sorts

|  | flag_P1 | flag_P2 | flag_P3 |
| --- | --- | --- | --- |
| Resp 1 | TRUE | FALSE | FALSE |
| Resp 2 | TRUE | FALSE | FALSE |
| Resp 3 | TRUE | FALSE | FALSE |
| Resp 4 | FALSE | TRUE | FALSE |
| Resp 5 | FALSE | TRUE | FALSE |
| Resp 6 | TRUE | FALSE | FALSE |
| Resp 7 | FALSE | TRUE | FALSE |
| Resp 8 | TRUE | FALSE | FALSE |
| Resp 9 | FALSE | FALSE | TRUE |
| Resp 10 | FALSE | TRUE | FALSE |
| Resp 11 | FALSE | TRUE | FALSE |
| Resp 12 | TRUE | FALSE | FALSE |
| Resp 13 | TRUE | FALSE | FALSE |
| Resp 14 | FALSE | TRUE | FALSE |
| Resp 15 | FALSE | FALSE | TRUE |

Table B: Distinguishing and consensus statements

| Statements | Distinguishes and Consensus | p1_p2 | sig_p1_p2 | p1_p3 | sig_p1_p3 | p2_p3 | sig_p2_p3 |
| --- | --- | --- | --- | --- | --- | --- | --- |
| 1 | Distinguishes all | 1.410 | 6* | 3.109 | 6* | 1.699 | *** |
| 2 | Consensus | 0.188 |  | -0.092 |  | -0.280 |  |
| 3 |  | 0.780 | ** | 0.543 |  | -0.237 |  |
| 4 | Consensus | 0.149 |  | -0.268 |  | -0.417 |  |
| 5 | Consensus | 0.319 |  | 0.056 |  | -0.263 |  |
| 6 |  | -0.575 | * | 0.172 |  | 0.748 |  |
| 7 | Distinguishes f3 only | 0.050 |  | -1.769 | *** | -1.819 | *** |
| 8 | Distinguishes f1 only | 0.703 | ** | 0.752 | * | 0.049 |  |
| 9 | Consensus | -0.480 |  | -0.092 |  | 0.388 |  |
| 10 | Distinguishes f2 only | -1.446 | 6* | -0.259 |  | 1.187 | ** |
| 11 | Distinguishes f1 only | -0.605 | * | -0.767 | * | -0.162 |  |
| 12 |  | 0.999 | *** | 0.508 |  | -0.492 |  |
| 13 | Consensus | -0.054 |  | -0.638 |  | -0.584 |  |
| 14 | Distinguishes f3 only | 0.510 |  | -2.471 | 6* | -2.981 | 6* |
| 15 | Consensus | -0.097 |  | 0.434 |  | 0.531 |  |
| 16 | Consensus | 0.270 |  | 0.063 |  | -0.207 |  |
| 17 | Consensus | -0.079 |  | 0.612 |  | 0.691 |  |
| 18 | Consensus | 0.107 |  | -0.644 |  | -0.751 |  |
| 19 | Distinguishes f2 only | -1.734 | 6* | -0.733 |  | 1.002 | ** |
| 20 | Distinguishes f2 only | -1.029 | *** | 0.308 |  | 1.337 | *** |
| 21 |  | -0.547 | * | -0.255 |  | 0.292 |  |
| 22 | Consensus | -0.137 |  | 0.327 |  | 0.464 |  |
| 23 |  | 1.154 | *** | 0.742 |  | -0.412 |  |
| 24 | Consensus | 0.142 |  | 0.360 |  | 0.218 |  |


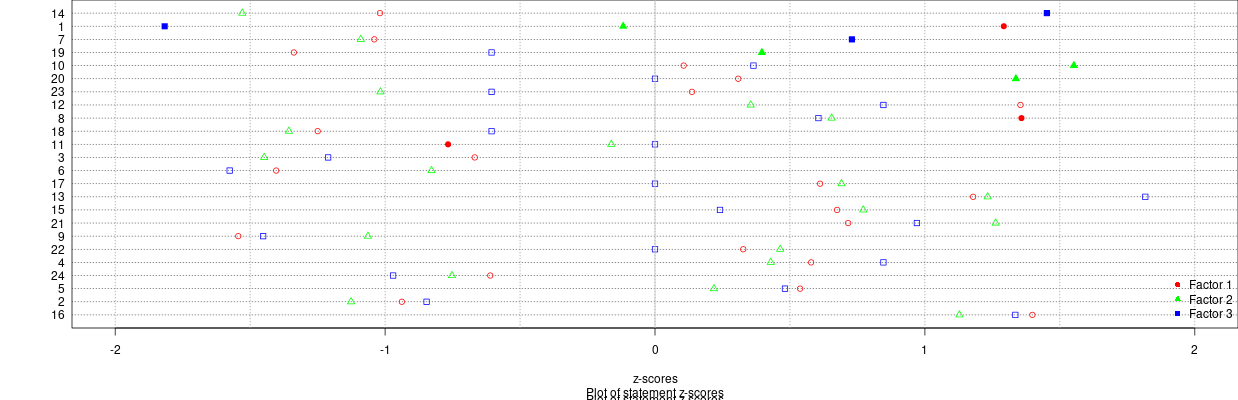


Figure A: Plot statement Z-scores


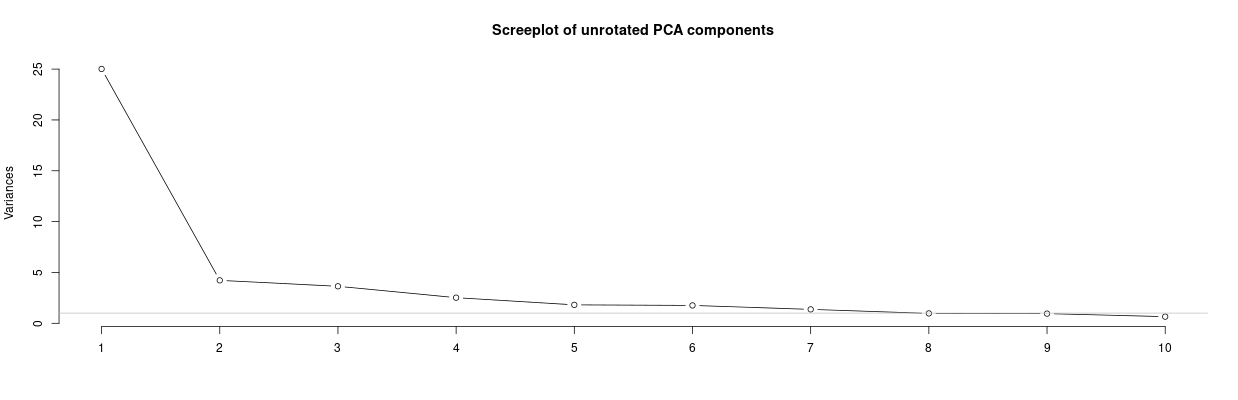


Figure B: Screeplot of unrotated components
